# Testing for baseline differences in randomized controlled trials: an unhealthy research behavior that is hard to eradicate

**DOI:** 10.1186/s12966-015-0162-z

**Published:** 2015-01-24

**Authors:** Michiel R de Boer, Wilma E Waterlander, Lothar DJ Kuijper, Ingrid HM Steenhuis, Jos WR Twisk

**Affiliations:** Department of Health Sciences and the EMGO Institute for Health and Care Research, Faculty of Earth and Life Sciences, VU University Amsterdam, De Boelelaan 1085, 1081 HV Amsterdam, The Netherlands; National Institute for Health Innovation, School of Population Health, The University of Auckland, Tamaki Campus, Private Bag 92019, Auckland Mail Centre, Auckland, 1142 New Zealand; Department of Epidemiology and Biostatistics, VU University Medical Centre, de Boelelaan 1118, Amsterdam, 1081 HV The Netherlands

**Keywords:** Epidemiology, Methods, Reporting, Nutrition behavior research, Statistical analyses

## Abstract

**Background:**

According to the CONSORT statement, significance testing of baseline differences in randomized controlled trials should not be performed. In fact, this practice has been discouraged by numerous authors throughout the last forty years. During that time span, reporting of baseline differences has substantially decreased in the leading general medical journals. Our own experience in the field of nutrition behavior research however, is that co-authors, reviewers and even editors are still very persistent in their demand for these tests. The aim of this paper is therefore to negate this demand by providing clear evidence as to why testing for baseline differences between intervention groups statistically is superfluous and why such results should not be published.

**Discussion:**

Testing for baseline differences is often propagated because of the belief that it shows whether randomization was successful and it identifies real or important differences between treatment arms that should be accounted for in the statistical analyses. Especially the latter argument is flawed, because it ignores the fact that the prognostic strength of a variable is also important when the interest is in adjustment for confounding. In addition, including prognostic variables as covariates can increase the precision of the effect estimate. This means that choosing covariates based on significance tests for baseline differences might lead to omissions of important covariates and, less importantly, to inclusion of irrelevant covariates in the analysis. We used data from four supermarket trials on the effects of pricing strategies on fruit and vegetables purchases, to show that results from fully adjusted analyses sometimes do appreciably differ from results from analyses adjusted for significant baseline differences only. We propose to adjust for known or anticipated important prognostic variables. These could or should be pre-specified in trial protocols. Subsequently, authors should report results from the fully adjusted as well as crude analyses, especially for dichotomous and time to event data.

**Summary:**

Based on our arguments, which were illustrated by our findings, we propose that journals in and outside the field of nutrition behavior actively adopt the CONSORT 2010 statement on this topic by not publishing significance tests for baseline differences anymore.

## Background

One of the recommendations of the CONSORT statement is that a table is presented showing baseline demographic and clinical characteristics for each group. Importantly, according to this recommendation in the CONSORT statement, “significance testing of baseline differences in randomized controlled trials (RCTs) should not be performed, because it is superfluous and can mislead investigators and their readers” [1]. In their paper on the explanation and elaboration of the CONSORT 2010 statement, the authors refer to an article of Altman dating back as far as 1985 [2]. In fact, there have been numerous papers on the subject, some of which have been published even before Altman’s article, in which significance testing of baseline differences has been discouraged or condemned [3-7]. Throughout the years there have been several literature reviews on this topic [6-9]. All of these reviews focused on the leading general medical journals although not all on the same ones. These reviews show a decreasing trend in the reporting of tests for baseline differences from it being an *“ubiquitous error”* in the 1978 and 79 issues of the NEJM [8] to 38.2% of the RCT’s published in the NEJM, the JAMA, the Lancet and the BMJ in the first half of 2007 [6].

In the last few years we have carried out several web-based and one real-life randomized controlled supermarket trials on the effect of several pricing strategies on food purchases. These have all been published in journals within the field of nutrition behavior, of which two in this journal [10-13]. In the process of writing and submitting these papers, we were, to some surprise, challenged by co-authors to include p-values of tests for baseline differences. The discussions were resolved and we were initially able to convince all authors of the fact that it would be better not to include these tests. This means that in our submitted papers we followed the CONSORT statement in not testing for baseline differences. However, after submission of the papers we were again faced with comments that tests of baseline differences should be added, but now from reviewers or even editors. To our surprise and dismay, these reviewers insisted on this point even after we had provided a logical explanation why we preferred not to present these p-values. Eventually, we decided to add the tests and as a result they are included in all four of our publications.

After our initial frustration, we started to think about possible explanations underlying the strong belief in testing for baseline differences in this field. One explanation could be that people, including scientists, are copycats and that they believe in what they see others do. An alternative, potentially related reason might be that most of the literature on this topic has been published in bio-statistical or methodological journals and is thus obscured from most of the applied behavioral nutrition or physical activity researchers.

The aim of this paper is therefore to negate the misconception that still exists among a large number of researchers in this field that baseline differences between intervention groups should be tested statistically by providing a strong argument as to why this is unnecessary. Furthermore, we argue that the choice of adjustment variables to include in the analysis should not be based on these tests. We will attempt to accomplish this by discussing the origin of the misconception and why statistical testing of baseline differences is flawed and misleading. Moreover, we will discuss practical consequences of testing for baseline differences and finally provide some guidance on how baseline differences should be taken into account. Most of our arguments are not new, but are restatements of what other authors have argued. Where possible we will use examples of the trials we published to illustrate these arguments.

## Discussion

### Why do people believe testing of baseline differences should be done and why is this a misconception?

The arguments most often used to substantiate the choice for statistical testing for baseline differences are that one needs to examine whether randomization was successful and that one needs to assess whether observed differences in baseline characteristics are ‘real’ or ‘important’. To test whether randomization was successful is quite problematic and, more importantly, not necessary [2,14]. First, it is problematic because there is no clear cut-off to decide when baseline differences are in concordance with proper randomization. One should remember that when any null-hypothesis is true, there is a probability of α (usually 0.05) that we will incorrectly reject it. In case of perfectly random allocation to conditions in trials, we would expect one out of 20 tests for significance to produce such a wrong result. However, what do we conclude when the proportion of such results exceeds α? For example, we found one statistically significant difference between the intervention and control condition out of the twelve baseline characteristics we tested (proportion = 0.08) in our web-based supermarket trial on the effects of a 25% discount on fruits and vegetables [10]. Does this mean that randomization was not performed properly? The simple answer is that we do not have any substantial numerical indication to that, because we only tested 12 differences and by chance could have found that 8% of these tests were statistically significant. In fact, a binomial test will show that, when we assume that all of these 12 tests involved independent true null-hypotheses (which they are not), the probability of encountering one statistically significant difference is 0.34. Our example is not an extreme. Usually only a limited number of baseline characteristics are tested [7]. Second, and more importantly, testing whether the randomization was performed properly is unnecessary. For one , the methods section of a paper should inform the reader whether randomization was performed properly and no statistical test will add any information about the correctness of this very procedure. Altman expressed this as *“performing a significance test to compare baseline variables is to assess the probability of something having occurred by chance when we know that it did occur by chance”* [2]. In other words: perfect randomization means we are testing between two samples from the same population by definition, and any extraordinary resulting p-value must be considered to be a random artefact. A second argument is that the most crucial fact to find out is not whether randomization was performed properly, but more if and how any possible baseline differences should be taken into account [3]. This is usually where the second argument for testing for baseline differences (the need to assess whether observed differences are real or important) comes into play.

**Figure 1:**
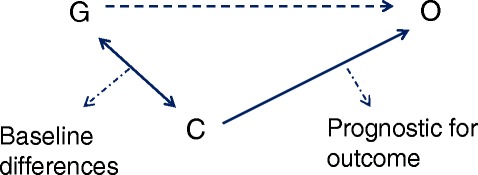
**Schematic representation of confounding factors in parallel arm randomized controlled trials.** G = group allocation; O = outcome; C = covariate.

The misconception in testing for baseline differences to determine whether real or important differences occur, should be seen in light of the difference between meaningful and statistically significant (detectable) differences. Meaningful differences are often not detected in a statistically significant manner [3,8,15], because trials are not powered for this purpose (type II error). On the other hand a statistically significant difference is not necessarily a meaningful one [3,14]. Meaningful differences here refer to differences in baseline characteristics that influence (confound) the results of a trial. Confounding occurs when there is a relation between a certain characteristic or covariate (C) and group allocation (G) and also between this characteristic and the outcome (O) [3,8,14], see Figure 1. In other words, confounding occurs when there is a difference between the intervention and control groups in a certain characteristic that is prognostic for the outcome of that trial. The amount of potential confounding does not depend upon whether these relations are statistically significant, but rather on the magnitude of these relations [3,8,15]. This could for example mean that a relatively small and non-statistically significant difference in a very strong prognostic factor could cause meaningful confounding and vice versa that a large difference in a characteristic unrelated to outcome would cause no confounding at all. Hence, potential confounders should not be chosen based on statistical tests of baseline differences.

### Practical consequences of testing for baseline differences

As discussed in the above, the practical consequences of testing for baseline differences mainly boil down to the question of whether the study conclusions would differ if only those covariates which proved to be significantly different at baseline were adjusted for in the analysis instead of adjusting for all prognostic factors.

In their review of 50 clinical trials in four major medical journals from 1997, Pocock et al [7] found that there were hardly any differences between estimates, CI’s and p-values from adjusted and unadjusted analyses. However, many authors have argued that the choice of covariates *can* potentially impact on the results from a trial [2,3,8,14]. With regard to the effect estimate this is especially true for smaller trials in which large baseline differences in prognostic variables usually go undetected. In- or excluding such prognostic variables as a covariate in/from the analysis can thus result in appreciably different effect estimates for the treatment effect. If the interest is in p-values and the null-hypothesis test regarding the treatment effect, the choice of covariates will have the same impact regardless of the sample size [4,14]. To illustrate this; let us assume that a certain difference in a given prognostic factor, say factor X, between intervention and control group results in a p-value of 0.15 after testing. If this p-value was derived in a small trial, the difference in this factor between groups would be relatively large, whereas for a large trial on the same treatment effect, this difference would be relatively small. Now, let us bear in mind that the p-value can be determined via dividing the effect estimate over the standard error of the effect estimate and also that the standard error decreases with an increasing sample size. Returning to our hypothetical example, we can argue that including factor X in the analysis of the treatment effect of the small trial, will influence the effect estimate of the treatment effect and by that also the p-value from the statistical test. In addition, including factor X in the analysis of the large trial, will have a smaller influence on the effect estimate of the treatment effect, but will have the same effect on the p-value as in the smaller trial. The latter is caused by the fact that the standard error from the treatment effect in the larger trial is smaller and therefore a relatively smaller change in the effect estimate after adjustment of prognostic factors, will have the same influence on the fraction of effect estimate and standard error and the resulting p-value.

We examined for the four trials we published [10-13] whether analysis adjusted for pre-defined potential confounders (referred to as fully adjusted models), which were reported in the publications, differed from analyses adjusted only for statistically different baseline characteristics with respect to the hypothesis tests and the magnitude of differences between conditions, see Table 1. We confined ourselves to outcomes related to fruit and/or vegetable purchases which were the primary outcomes analyzed in these studies. For three of the trials (study 2,3 and 4) we did not observe any statistically significant baseline differences, which means that we compared our fully adjusted models with crude models. For the other trial (study 1), we found one statistically significant difference, namely for level of education. For that trial we therefore compared results from the fully adjusted analyses (including adjustment for level of education) with those from analyses adjusted for level of education only. In these four trials a multitude of effects were analyzed, i.e. for different comparisons of several conditions and/or for different time points. The different conditions that were compared were: regular prices versus 25% discount on fruits and vegetables (study 1); combinations of price reductions on healthy foods (no; 25%; 50%) and price increases on unhealthy foods (5%; 10%; 25%) (study 2); 50% price discounts on fruits and vegetables, nutrition education, 50% price discounts plus nutrition education, or no intervention (study 3); combinations of price reduction (10%; 25%; and 50%) an different labels (‘special offer’, ‘healthy choice’ and ‘special offer & healthy choice’) (study 4).

**Table 1:** **Results from four randomized controlled supermarket trials with different methods of adjustment for baseline covariates**

**Study**	**Comparisons**	**Adjustment for covariates**	**Outcome**	**B**	**Lower 95% CI**	**Upper 95% CI**	**p-value**
1. Effects of 25% discounts on fruits and vegetables in a web-based supermarket [10]	25% discount on fruits and vegetables versus regular prices	Adjusted for statistically significant characteristics only (= level of education)	Fruit (g)	126	−376	630	0.62
			Vegetables (g)	445	−105	995	0.11
			F&V (g)	571	−300	1443	0.20
		Fully adjusted (including for level of education)	Fruit (g)	481	−69	1,030	0.09
			Vegetables (g)	504	−64	1,071	0.08
			F&V( g)	984	97	1,872	0.03
2. Introducing taxes, subsidies or both in a web-based supermarket [11]	25% discount on healthy foods versus no discount	Adjusted for statistically significant characteristics only (= none)	Fruit (g)	−376	−1097	345	0.03*
			Vegetables (g)	88	−678	854	0.01*
		Fully adjusted	Fruit (g)	−382	−1105	341	0.10*
			Vegetables (g)	52	−665	769	0.05*
	50% discount on healthy foods versus no discount	Adjusted for statistically significant characteristics only (= none)	Fruit (g)	599	−126	1,323	0.03*
			Vegetables (g)	1034	264	1,804	0.01*
		Fully adjusted	Fruit (g)	420	−322	1,163	0.10*
			Vegetables (g)	821	85	1,556	0.05*
	10% versus 5% increase on unhealthy foods	Adjusted for statistically significant characteristics only (= none)	Fruit (g)	216	−500	933	0.83*
			Vegetables (g)	−128	−889	633	0.45*
		Fully adjusted	Fruit (g)	304	−421	1,029	0.69*
			Vegetables (g)	121	−598	840	0.59*
	25% versus 5% increase on unhealthy foods	Adjusted for statistically significant characteristics only (= none)	Fruit (g)	82	−643	807	0.83*
			Vegetables (g)	345	−425	1,115	0.45*
		Fully adjusted	Fruit (g)	83	−646	813	0.69*
			Vegetables (g)	368	−355	1,091	0.59*
3. The effects of price discounts on fruits and vegetables with or without health education in a real supermarket [12]	50% discount on fruits and vegetables versus no intervention	Adjusted for statistically significant characteristics only (= none)	F&V(g) at 1 month	2386	87	4,685	0.04
			F&V (g) at 3 months	1,226	−1,063	3,596	0.31
			F&V (g) at 6 months	5,252	2,836	7,668	<0.01
			F&V (g) at 9 months	−826	−3,284	1,632	0.51
		Fully adjusted	F&V(g) at 1 month	1,295	−1,031	3,621	0.28
			F&V (g) at 3 months	951	−1,293	3,194	0.41
			F&V (g) at 6 months	3,894	1,500	6,287	<0.01
			F&V (g) at 9 months	−1,397	−3,833	1,038	0.26
	Nutrition education versus no intervention	Adjusted for statistically significant characteristics only (= none)	F&V(g) at 1 month	−1,583	−4,049	884	0.21
			F&V (g) at 3 months	−886	−3,432	1,661	0.50
			F&V (g) at 6 months	35	−2,571	2,640	0.98
			F&V (g) at 9 months	−1,235	−3,747	1,277	0.34
		Fully adjusted	F&V(g) at 1 month	−913	−3,383	1,557	0.47
			F&V (g) at 3 months	−91	−2,584	2,402	0.94
			F&V (g) at 6 months	1,075	−1,468	3,617	0.41
			F&V (g) at 9 months	−481	−2,937	1,976	0.70
	50% discount on fruits and vegetables + Nutrition education versus no intervention	Adjusted for statistically significant characteristics only (= none)	F&V(g) at 1 month	1,912	−354	4,179	0.10
			F&V (g) at 3 months	978	−1,386	3,343	0.42
			F&V (g) at 6 months	5,383	2,958	7,808	<0.01
			F&V (g) at 9 months	−1,176	−3,582	1,230	0.34
		Fully adjusted	F&V(g) at 1 month	1,290	−1008	3,587	0.27
			F&V (g) at 3 months	1,213	−1,078	3,504	0.30
			F&V (g) at 6 months	5,556	3,188	7,925	<0.01
			F&V (g) at 9 months	−1,157	−3,533	1,220	0.34
4. Effects of different discount levels on healthy products coupled with a healthy choice label in a web-based supermarket [13]	25% versus 10% discount on healthy foods	Adjusted for statistically significant characteristics only (= none)	Fruit (g)	398	−384	1,180	0.33*
			Vegetables (g)	−83	−887	721	<0.01*
		Fully adjusted	Fruit (g)	393	−442	1,228	0.52*
			Vegetables (g)	39	−808	885	0.02*
	50% versus 10% discount on healthy foods	Adjusted for statistically significant characteristics only (= none)	Fruit (g)	544	−193	1,280	0.33*
			Vegetables (g)	1,108	350	1866	<0.01*
		Fully adjusted	Fruit (g)	410	−361	1,182	0.52*
			Vegetables (g)	1,017	226	1,807	0.02*
	Special offer label versus combined label	Adjusted for statistically significant characteristics only (= none)	Fruit (g)	61	−676	797	0.94*
			Vegetables (g)	−436	−1,193	322	0.52*
		Fully adjusted	Fruit (g)	74	−704	853	0.78*
			Vegetables (g)	−479	−1,286	328	0.50*
	Healthy choice label versus combined label	Adjusted for statistically significant characteristics only (= none)	Fruit (g)	−76	−858	705	0.94*
			Vegetables (g)	−217	−1,021	587	0.52*
		Fully adjusted	Fruit (g)	−200	−1,014	613	0.78*
			Vegetables (g)	−282	−1,138	574	0.50*

*Overall p-value comparing the three groups.

CI = confidence interval; F&V = fruit and vegetables.

For study 2 and 4, two web-based supermarket trials, there were no appreciable differences for any of the observed effect sizes between crude and fully adjusted analyses. However, in study 2 the statistical tests from the crude analyses on the effects of discounts did result in statistically significant findings, whereas the adjusted did not (crude p-values of 0.03 and 0.01 versus adjusted p-values of 0.10 and 0.05). The conclusions of the paper were based on the results from the crude and fully adjusted models and also on the results from other endpoints such as number of healthy foods and total number of items purchased. For study 1, a web-based supermarket trial on the effect of 25% discounts on fruits and vegetables, the fully adjusted analyses resulted in larger differences compared to the analyses adjusted only for level of education. In addition, for combined fruits and vegetables (F&V) purchases the difference between the 25% discount condition and the control condition was statistically significant in the adjusted analysis (984 grams; 95% CI: 97, 1,872; p = 0.03), but not in the analysis adjusted for level of education only (571 grams; 95% CI: −300, 1,443: p = 0.20). Analyses of fruit and vegetables separately did not show any statistically significant differences at the 5% significance level irrespective of the adjustments made. The conclusion of the article was primarily based upon the results from the fully adjusted analysis. Therefore conclusions might have very well been different had the analyses been adjusted for statistically significant baseline differences only. Study 3 comprised a real supermarket trial on the effects of price discounts and nutrition education and the combination of both. Price discounts and price discounts in combination with nutrition education showed statistically significant effects compared to no intervention at six months in the crude and fully adjusted analyses. The magnitude of the effect was somewhat attenuated in the fully adjusted analysis for the effect of price discounts (5,252 vs. 3,894 g per 2wks), but not for the combined effect of price discounts and nutrition education (5,383 vs. 5,556 g per 2wks). No other statistically significant effects were observed, except for the crude effect of price discounts at one month (2386 g per 2 wks; 95% CI: 87, 4,685: p =0.04). This effect was much smaller and not statistically significant in the adjusted analysis (1,295 g per 2 wks; 95% CI: −1,031, 3,621: p =0.28). Conclusions were restricted to the statistically significant and fairly consistent results at 6 months follow-up.

To summarize our examples, only one out of the four studies did not show differences between fully adjusted analyses and analyses adjusted for statistically significant baseline differences only. Two studies showed some differences, either in results from statistical tests or also in actual effect sizes, but these differences most likely would not have affected the conclusions, whereas one study showed differences that could very well have affected the conclusions.

We realize that we have only examined four trials and that results from comparisons for these specific trials are not generalizable with respect to the number, amount and precipitated relevance of differences we observed. Nevertheless, these examples show that using all the available relevant information in the analyses of trials might result in different outcomes compared to analyses where covariates are restricted to statistically significant, but not necessarily important, differences between groups at baseline. In the following we will discuss these practices in some more detail and we will try to provide some practical guidelines on how to deal with adjustment for covariates in trials alternative to the practice of adjusting only for statistically significant baseline differences.

### Some practical guidance for dealing with baseline differences

One could argue that analyses from trials that have been randomized adequately need not be adjusted at all, because the analysis will result in a valid estimate of the treatment effect [14]. This is based on the notion that when one would endlessly repeat a trial on a specific comparison (same treatment and control condition and same numbers in both groups) and one would average over all effect estimates, that this would result in the ‘true’ treatment effect. However, this is only a theoretical notion and the ‘true’ treatment effect remains unknown in practice. This means that a given trial may be close to the ‘true’ treatment effect or it might be further away. Imbalance in a trial will mean that the effect estimate of that trial will generally be further away from the ‘true’ treatment effect. An epidemiologist would say that the internal validity is compromised. By adjusting for known prognostic variables that differ, be it statistically significant or not, between the treatment groups, the effect estimate will be closer to the ‘true’ effect or in other words: it will be more internally valid. Another or related advantage of adjusting for known prognostic variables, which also applies when there is no difference at baseline, is that the effect estimate will be more precise, i.e. the confidence interval will usually be smaller [14,16]. The reason for this is that the prognostic factors will explain part of the unexplained variance which reduces the standard error of the treatment effect. This is in fact the whole idea behind analyses of covariance (ANCOVA). We therefore agree with others [2-4,14,17] that adjustment for prognostic variables should be made. This is irrespective of the fact whether differences on these variables were statistically significant at baseline.

Some authors have argued that prognostic factors may not be known beforehand [6,7]. We do not think this is a major problem however, because, as put so eloquently by Senn [4] “*Researchers should remember that we are often studying new therapies, but we more rarely find ourselves studying new diseases”,* meaning that prognostic variables often can be extracted from previous studies. An additional worry that has been expressed, is that the choice of prognostic variables as covariates in the analysis model can influence the results [7]. There are situations where adjusting for one prognostic factor might result in a statistically significant finding while adjusting for a different one would not, as was illustrated in our example. Some authors might in those cases be tempted to specifically adjust for those covariates that lead to statistically significant results which they subsequently report in their papers. We agree that this can pose a problem for a given trial. However, there is a straightforward solution to this problem. Most journals nowadays demand that authors include all primary and secondary endpoints in the protocols submitted to a trial register. It would be simple to also request mentioning the covariates here. This would also be feasible from a researcher’s perspective, because researchers, naturally, have to take important prognostic factors into consideration before starting the trial so that they can assess these variables at baseline. We acknowledge that there are some pitfalls in practice, for example the fact that trial protocols do not have to be registered before the trial starts and others may (therefore) have different views on how to tackle potential problems relating to the (selective) choice of prognostic factors.

Our discussion of the adjustment for baseline variables from RCT´s in the above is limited to direct adjustment of variables in general linear models, i.e. when the outcome is continuous. It should be noted that there are alternative methods of dealing with imbalances between groups such as pre-stratification or minimization, which we will not dwell on any further in this paper. In addition, when outcomes are dichotomous or time to event, i.e. when logistic regression or survival analyses are used, things are more complex. In contrast to mean differences (MD’s), odds ratio’s (OR’s) and hazard ratio’s (HR’s)are non-collapsible effect estimators [18]. This means that for trials on a specific comparison, the crude effect estimate will on average coincide with the adjusted effect estimate when analyzing MD’s, but not when analyzing OR’s or HR’s [6,18]. For these latter effect estimates, the crude (also sometimes referred to as marginal or population averaged) effects will be systematically closer to the null value than the adjusted (also sometimes referred to as subject specific) effects. This means that crude and adjusted effect estimates have different interpretations when analyzing OR’s or HR’s [6,17]. At the moment there seems to be no consensus as to which estimate (crude or adjusted) is more informative from a clinical or policy perspective [6]. Therefore, we agree with others [6], that especially for binary and time to event outcomes, results from RCT’s should be presented for crude as well as adjusted analyses. In that way, at least the results from the crude analyses regarding trials on similar comparisons are comparable.

### Summary

We have discussed that testing for baseline differences serves no purpose and can be misleading, especially because some researchers still think that these tests are the basis for choosing covariates in their analyses. This practice however ignores the prognostic strength of covariates, which is a more important characteristic to take into account, because adjustment for prognostic factors can also increase precision of effect estimates. We have shown in our data from four supermarket trials that the choice of covariates is not a trivial matter and that effect estimates could appreciably differ between strategies. We therefore propose to include and register covariates in trial protocols, to register these protocols before the start of the trial and to publish fully adjusted as well as crude analyses results. Finally, based on these arguments, we propose that journals in and outside the field of nutrition behavior actively adopt the CONSORT 2010 statement on this topic by not publishing these tests anymore.
